# The Impact of Cardiovascular Rehabilitation on Psychophysiological Stress, Personality and Tryptophan Metabolism: A Randomized Pilot Feasibility Study

**DOI:** 10.3390/antiox10091425

**Published:** 2021-09-07

**Authors:** Jolana Wagner-Skacel, Sabrina Mörkl, Nina Dalkner, Frederike Fellendorf, Werner Fitz, Bianca Brix, Ruslan Neshev, Sarah Wedenig, Petra Mächler, Andreas Dorr, Rainer Picha, Maximilian E. Rudlof, Till O. Bartel, Josef M. Tatschl, Johanna M. Gostner, Susanne A. Bengesser, Eva Z. Reininghaus, Josef Jenewein, Nandu Goswami

**Affiliations:** 1 Department of Medical Psychology and Psychotherapy, Medical University Graz, 8036 Graz, Austria; jolana.wagner-skacel@medunigraz.at (J.W.-S.); Josef.Jenewein@medunigraz.at (J.J.); 2 Department of Psychiatry and Psychotherapeutic Medicine, Medical University Graz, 8036 Graz, Austria; nina.dalkner@medunigraz.at (N.D.); frederike.fellendorf@medunigraz.at (F.F.); WernerJosef.Fitz@uniklinikum.kages.at (W.F.); sarah.wedenig@stud.medunigraz.at (S.W.); susanne.bengesser@medunigraz.at (S.A.B.); eva.reininghaus@medunigraz.at (E.Z.R.); 3 Department of Physiology, Otto Loewi Research Center for Vascular Biology, Immunology and Inflammation, 8010 Graz, Austria; bianca.brix@medunigraz.at (B.B.); ruslan.neshev@medunigraz.at (R.N.); maximilian.rudlof@stud.medunigraz.at (M.E.R.); till.bartel@stud.medunigraz.at (T.O.B.); nandu.goswami@medunigraz.at (N.G.); 4 Rehabilitation Center for Cardiovascular Disease, 8061 St. Radegund, Austria; drmaechler@hotmail.com (P.M.); andreas.dorr@pv.at (A.D.); rainerpicha@gmail.com (R.P.); 5 Health Psychology Unit, Institute of Psychology, Karl-Franzens University of Graz, 8010 Graz, Austria; mail@joseftatschl.com; 6 Biochemical Immunotoxicology Group, Institute of Medical Biochemistry, Medical University Innsbruck, 6020 Innsbruck, Austria; johanna.gostner@i-med.ac.at

**Keywords:** cardiovascular rehabilitation, cortisol, kynurenine totryptophan ratio, personality functioning, sleep, psychosocial wellbeing

## Abstract

Multicomponent cardiac rehabilitation (CR) is a secondary prevention strategy for cardiac patients to tackle stress and psychosocial wellbeing. However, there is a lack of data on its psychoneuroimmunological effects and of biomarkers to determine individual risk and to develop treatment strategies. We conducted a pilot randomized controlled trial (RCT) to investigate the feasibility of deriving psychophysiological stress markers in patients with cardiovascular diseases. Thirty individuals with cardiovascular disease (mean age 58.8 years; 23.3% female) were enrolled and randomized into three treatment groups: standard rehabilitation, yoga, or transcendental meditation (TM). Depression, anxiety, sleep, stress perception, personality functioning, hair cortisol, serum tryptophan, kynurenine and neopterin concentrations were estimated at baseline and after a four-week intervention. Hair cortisol levels decreased significantly after rehabilitation in all groups *(F* = 15.98, *p* < 0.001). In addition, personality functioning improved in all patients over time. Participants with impairments in personality functioning showed a positive correlation with baseline neopterin that did not remain significant after Bonferroni correction. Concentrations of serum tryptophan and its metabolite kynurenine did not change significantly. This pilot RCT provides preliminary evidence of multicomponent CR leading to stabilization of hair cortisol levels and improved psychophysiological wellbeing and personality functioning. Impairments in personality functioning were correlated with neopterin levels, which may impact the symptomatology and outcome.

## 1. Introduction

Cardiovascular diseases (CVD) represent one of the leading causes of morbidity and mortality worldwide [1]. Physiological and environmental stressors trigger inflammatory cascades that increase the risk of atherosclerosis and insulin resistance, and thus the development of cardiovascular diseases (CVD) [2]. Psychocardiologically, the onset and course of CVD are based on a biopsychological model consisting of an individual genetic makeup in interaction with interpersonal experiences during childhood and adolescence, and various environmental stressors [3]. Environmental and behavioral factors such as smoking, poor diet, inactivity and preventable exposure to stress, play a significant role and contribute to the individual risk [4]. The need to implement psychosocial treatment strategies is well-founded by the robust evidence of psychosocial factors in cardiological care. Until now, biomarkers to derive prognostic effects of psychosocial interventions are not well investigated [5].

In heart disease, the biological, psychological and cognitive response to chronic stress acts either as a trigger or an independent factor that impacts cardiac outcomes. In contrast to this, cardiac rehabilitation is the main factor that appears to manage psychological disorders in CVD patients, helping them to reduce the bidirectional effects of stress on their minds and bodies [6].

The prevalence of major depressive disorders in individuals with CVD is approximately twice as high as in the general population [7]. In this context, the role of inflammation and its influence on mortality has often been discussed [8]. The regulation of tryptophan (Trp) concentration, necessary for systemic homeostasis, integrates essential pathways involved in metabolic stress response, immunity and neuroactivation. The psychocardiac and neuroimmunological consequences of Trp metabolism and the susceptibility of this pathway to modulation by lifestyle-related factors have important implications for developing both diagnosis and treatment options [9]. For example, an increase in immune-stimulated synthesis of kynurenine (Kyn) from Trp by indoleamine 2,3-dioxygenase (IDO-1), leading to a higher Kyn to Trp ratio, has been observed in patients with CVD in association with the development of depressive symptoms [10]. Trp belongs to the essential amino acids. It is metabolized to a number of downstream products such as serotonin, melatonin, tryptamine, Kyn, kynurenic acid, 3-hydroxykynurenine, quinolinic and picolinic acid [11]. A decreased antioxidant status in patients with CVD corresponded with an increased cellular inflammatory response with elevated oxidative stress as indicated by higher neopterin concentrations [12]. A shift towards the Kyn pathway is common in situations of increased oxidative stress, chronic inflammation and often associated with elevated cortisol levels based on an activated hypothalamic-pituitary-adrenal (HPA) axis, which is linked to mood disorders.

Personality and binding structures affect CVD via the stress axis. Insecure attachment is linked to heightened stress reactivity and is significantly linked to primary hypertension [13]. Experiencing stress in early childhood increases the risk for mental, cardiovascular and metabolic illnesses, along with higher mortality rates [14]. Personality functioning and personality structure describe enduring maladaptive patterns of emotion, cognition, regulation and behavior. Patients suffering from an impairment in personality functioning have difficulties in interpersonal relationships as well as difficulties in self-regulation and personality traits [15]. When stressed by external and internal conflicts, problems in flexible functioning lead to adverse health behaviors and interpersonal difficulties [16]. Via stimulation of stress-regulating systems like the HPA axis or the autonomic nervous system, chronic activation results in dysregulation. It may finally lead to aggregate physiological consequences.

Sleep quality is known to modify cardiovascular regulation. CVD is associated with alterations of physiological sleep. Sleep disorders can significantly alter the cardiovascular system [17]. Human and animal sleep deprivation studies have reported the association of sleep disturbances with altered clock gene expression. This altered clock gene expression vitally affects neurobiological responses to stress [18]. Chrono-disruption sensitizes individuals to stress and increases their vulnerability associated with objective or subjective lack of social support as well as a higher risk of developing cardiovascular diseases and a higher risk of mortality [19]. Aside from disturbed circadian rhythms, multicomponent rehabilitation can improve functional capacity and quality of life in patients with CVD [20]. Individually tailored therapy with special consideration of psychophysiological stress reduction and sleep quality is needed to cope with everyday life challenges after rehabilitation.

The current investigation aimed at analyzing the beneficial effects of cardiac rehabilitation on psychosocial wellbeing and its biological underpinnings. More specifically, this study investigated changes in psychophysiological stress markers in patients with CVD after stress-reducing interventions such as yoga or transcendental meditation (TM) additionally to the standard rehabilitation program, or standard rehabilitation only (treatment-as-usual) in a four-week rehabilitation program. Individual differences in the emotional, immunological and physiological response to a rehabilitation program may be critical for both the predictive evidence and the construction of new management models and novel treatment strategies. Thus, we conducted a single-institution clinical trial and randomly assigned patients into three groups (Yoga, TM, Standard) to address the following questions:

1. Is there a significant difference in psychological variables (depression, anxiety, somatization, psychological wellbeing and sleep quality) before and after the intervention and between the three groups?
2. Is there a significant difference in stress markers (hair cortisol, Kyn/Trp ratio) between the groups Yoga, TM and standard after the intervention?
3. Is there an association between personality functioning and stress markers (hair cortisol, Trp, Kyn, neopterin, Kyn/Trp ratio) at baseline?

We hypothesized that patients, independent of the intervention (Yoga, TM, or standard), may report fewer psychological symptoms (anxiety, depression, somatization) and less sleep disturbances after the intervention, which is also reflected in significant changes in biomarkers (cortisol, neopterin, kyn/trp ratio) indicating reduced inflammatory processes.

In addition, we hypothesized that participants would show increased stress and immune activation markers and an impairment in personality structure, which includes the capacities for self and object recognition, regulation, communication and attachment.

## 2. Materials and Methods

### 2.1. Study Participants

Data collection (T1) began in October 2019 and ended (T2) in November 2019 after a four-week rehabilitation program. The study was approved by the Ethics Committee of the Medical University Graz (approval number EK 31-443 ex 18/19) and was conducted according to the Declaration of Helsinki. This study was registered in the NCT Clinical Trial Registry (registration number: NCT5035758).

Patients, aged between 40 and 80 years, admitted to the Cardiac Rehabilitation Centre in St. Radegund, Styria, Austria after myocardial infarction (MI), ST-level myocardial infarction (STEMI) or non-ST-level myocardial infarction (NSTEMI), acute coronary syndrome (ACS), coronary artery disease (CAD) with percutaneous coronary intervention (PCI), or after coronary artery bypass graft (CABG) were included. On average, cardiac rehabilitation was commenced four weeks after the cardiac event.

Patients who needed to be monitored because of clinical symptoms such as grade III heart failure with a mini-mental score below 26, or who were not sufficiently mobilized to perform yoga exercises or mediation techniques, were excluded.

A total of 30 patients (seven females) with a mean age of 58.8 years (SD = 9.8) participated in this study. After recruitment, participants were randomly assigned into three groups using an online randomizing tool (www.random.org). The treatment-as-usual, **Standard group**, received the standard exercise therapy of the rehabilitation center. The intervention **TM** **group** received transcendental meditation (TM) sessions additionally to the standard exercise therapy. The **Yoga** **group** received yoga sessions additionally to the standard exercise therapy. Patients could withdraw from the study at any moment without providing any justification.

### 2.2. Study Intervention

All groups followed the four-week standard rehabilitation exercise program which consisted of an individual exercise schedule (depending on the performance during spiro-ergometry on the first day), physiotherapy sessions (e.g., massages, lymphatic drainages), different lifestyle education seminars (on smoking, nutrition, weight management), psychological assistance and medical check-ups if necessary (e.g., blood analysis, X-rays, ergometry, sonography, 24-h ECG and 24-h blood pressure). The following exercise options were available: bicycle, treadmill, walking, hiking (in groups), gymnastics, strength endurance workouts, including weight-lifting and swimming. Participants in the two intervention groups (Yoga and TM) attended the regular rehabilitation program and received additional yoga or meditation sessions. Each yoga and meditation session lasted for 20 min and was conducted twice a day (between 7:00–7:30 a.m. and 17:00–17:30 p.m.), seven times per week. The meditation group was instructed on the technique of transcendental meditation (TM) by professional meditation teachers of the “Austrian society of Maharishi Vedic sciences” (Österreichische Gesellschaft für Maharishi Vedische Wissenschaft) in a four-day seminar. Afterwards, participants were able to perform the meditation on their own. Two rooms in the clinic were available every morning and evening to provide them a quiet place to meditate. The Yoga group received guided yoga classes by professional yoga teachers in the clinic’s gym. The yoga program consisted of various yoga techniques like Asanas (gentle movement exercises), Body-Scan (a type of mindful meditation), Pranayamas (breathing techniques) and affirmations. The data were collected within a pilot study of the Otto Loewi Research Center for Vascular Biology, Immunology and Inflammation (Graz, Austria).

### 2.3. Description of the Psychological Tests

#### Psychological Assessment

**The Beck Depression Inventory** (BDI-II) with 21 items was used as a self-evaluation report to assess the severity of depressive symptoms with a total score ranging from 0 to 63 points. A score below 18 indicated a lack of clinical depression. The scale has been shown to demonstrate an internal consistency with a Cronbach’s α ≥ 0.84 and reliability of *r* ≥ 0.75 [21]. 

For the **level of personality functioning,** we used the short version of the OPD Structure Questionnaire (OPD-SQS), a viable screening instrument for treatment planning and therapy focus [22]. The OPD-SQS consists of 12 items with three subscales (self-perception, contact, relationship). The subscale ‘self-perception’ shows aspects of the self with structural skills of emotion regulation. The subscale ‘contact’ demonstrated interactional skills with aspects of self-uncertainty. The subscale ‘relationship’ represented relationship experiences and connections to expectations of new relationships. The score reaches from 0 (‘highest structural level’) to 48 (‘lowest structural level’) with an internal consistencies range from α = 0.87 to 0.89 [22]. 

The “**Aktuelle Stimmungsskala**” (ASTS) was used to assess current mood state. It is a shortened version of the ‘profile of mood state scale’ (POMS) including 19 adjectives. Patients estimate how well an adjective reflects their current feelings on a scale ranging from seven (very strong) to one (not at all) summarized on five different scales. The items can be summed up separately to form five dimensions: sadness, hopelessness, positive mood, tiredness, and anger. Higher scores in a sub-core indicate a higher expression of the respective momentary feeling. Moreover, an overall measure can be formed to describe the current negative mood [23].

The **PSQ-20 Perceived Stress Questionnaire** 20 items version is the validated short version of the Perceived Stress Questionnaire, and its German version was used to assess subjectively experienced stress. Four different subscales can be derived from the PSQ-20: worries, tension, joy and demands. Additionally, all items can be added up to a total score. Higher scores within a subscore indicate a higher expression of the respective feeling, whereas a higher total score indicates a higher level of perceived stress in general [24].

The German version of the **PSQI Pittsburgh Sleep Quality Index** was used to evaluate sleep quality. It is a validated self-report survey consisting of 19 items (Range 0–3). These items assess relevant domains of sleep quality over one month and generate seven different subscores: subjective sleep quality, sleep latency, sleep duration, habitual sleep efficiency, sleep disturbances, use of sleeping medication, and daytime dysfunction. In addition to the subscores, the sum of scores yields one global score (Range 0–21). Healthy sleepers usually have a total value of less than five points [25].

The German version of the **Six-Item Short Form of the Spielberger State-Trait Anxiety Inventory (STAI-6)** was used to assess fluctuations in state anxiety (how anxious one feels at the moment). A total score can be calculated with six items. Higher scores indicate a higher level of state anxiety [26].

The German version of the **Short Form Health Survey 36 (SF-36)** was used to evaluate the subjective health of the patients. It is a self-rated questionnaire consisting of 36 items, wherein each item is either a scale itself or represents a part of a scale. The items vary from binary “yes-no” questions to six-level answer scales. The survey covers eight dimensions of subjective health on physical functioning and perceptions. For each subscale a score can be calculated (Range 0–100), whereby higher scores define a more favorable health state [27].

### 2.4. Molecular Biological Analyses

#### 2.4.1. Tryptophan and Kynurenine

An autosampler (model 400, both Varian ProStar, Palo Alto, CA, USA), an UV-spectrometric detector (SPD-6A, Shimadzu, Korneuburg, Austria), and a fluorescence detector (model 360, Varian ProStar, Palo Alto, CA, USA) were used to detect the amino acids (Trp, Kyn) in serum by high-performance liquid chromatography (HPLC) using a Varian ProStar HPLC system equipped with a solvent delivery module (model 210), as reported previously [28]. Sample preparation included protein precipitation with trichloroacetic acid. 3-nitro-L-tyrosine was used as an internal standard. Trp was measured by its native fluorescence (excitation wavelength of 286 nm, emission wavelength of 366 nm), Kyn and 3-nitro-L-tyrosine were detected at a wavelength of 360 nm. Neopterin concentrations were determined by an enzyme-linked immunosorbent assay (ELISA) (BRAHMS, Henningsdorf, Germany) with a sensitivity of 2 nmol/L neopterin. The Kyn to Trp ratio can be applied to indicate IDO-1 activity if paralleled by increased concentrations of proinflammatory mediators such as neopterin [29].

#### 2.4.2. Cortisol

Cortisol was measured in the hair of the patients (Cortisol ELISA 12 × 8 Tests, Nova Tec Immundiagnostica GmbH, 63128 Diezenbach, Germany). Older hair (2 cm long) contained the cortisol amount that represented the “pre-rehabilitation” level. The most recent, youngest hair (1 cm long) contained the amount of cortisol after rehabilitation. Therefore, the concentration of cortisol was measured at different lengths of hair samples taken from the participants. The two lengths were 2 cm and 1 cm, as normal human hair grows approximately 1 cm per month. Thus, the measurements taken at 2 cm corresponded to an earlier point in time (before the rehabilitation), and the one at 1 cm corresponded to the time directly after the rehabilitation.

### 2.5. Statistical Analyses 

All analyses were performed using the Statistical Package for Social Sciences (IBM SPSS 25.0, Armonk, NY, USA). Due to the small sample size of each group (*n* = 10) the Shapiro-Wilk test was used to test for normal distribution of the dependent variables. Group differences at baseline were tested either with nonparametric tests (Kruskal Wallis Test), robust analysis of variance (ANOVA) or multivariate analysis of variance (MANOVA).

Single-factor repeated measures analysis of variance (RM-ANOVA) were calculated to assess the statistical significance of differences between the three groups (Yoga vs. TM vs. Standard) at two points of measurement (pretest vs. posttest).

Within-group comparisons pretest vs. posttest were computed with paired sample *t*-test (if normal distribution was given) or the nonparametric Wilcoxon-Test. Correlations of parameters were calculated using Spearman’s correlations. *p*-values below 0.05 were considered statistically significant. Bonferroni-adjustment was done to correct for multiple comparisons (RM-ANOVAS: *p* < 0.007, *n* = 7 tests; correlation analyses: *p <* 0.0025, *n* = 20 tests).

## 3. Results

### 3.1. Normal Distribution

Trp (posttest), Kyn (pretest, posttest), Kyn/Trp (pretest, posttest) neopterin (pretest, posttest) were not normally distributed (*p* < 0.05) according to the KS and Shapiro Wilk test. 

### 3.2. Test for Homogeneity 

The Levene’s tests for all conducted RM- ANOVAs showed that the residuals were homogenously distributed (the Levene’s tests were not significant).

### 3.3. Descriptive Statistic (Desciption of the Sample)

The descriptive parameters are shown in Table 1. No significant group differences regarding the sex distribution could be observed (χ^2^(2) = 5.96, *p* = 0.051). Furthermore, a single factor variance analysis showed no significant differences between the groups in terms of average age (*F*(2,27) = 0.32, *p* = 0.729, *η_p_^2^* = 0.023), nor did a Kruskal-Wallis test for the follow-up duration (χ^2^(2) = 0.85, *p* = 0.640). (M)ANOVAs indicated that the groups did not differ in psychological test variables at pretest (BDI-II: *F*(6,6) = 2.03, *p* = 0.204, *η_p_^2^* = 0.670; ATST: *F*(10,44) = 1.05, *p* = 0.419, *η_p_^2^ =* 0.193; PSQ-20: *F*(8,42) = 1.12, *p* = 0.368, *η_p_^2^* = 0.176; PSQI: *F*(20,30) = 0.77, *p* = 0.726, *η_p_^2^* = 0.339; STAI: *F*(8,48) = 0.53, *p* = 0.204, *η_p_^2^* = 0.670; SF-36: *F*(6,6) = 2.03, *p* = 0.204, *η_p_^2^* = 0.670; OPD-SQS: *F*(6,6) = 2.03, *p* = 0.204, *η_p_^2^* = 0.670).

### 3.4. Group Differences (Standard vs. TM vs. Yoga Group)

#### 3.4.1. Intervention Effects in Psychological Variables

RM-ANOVAs with factor group (Yoga, TM and standard group) and dependent variables PSQI, STAI, OPD-SQS, SF-36, BDI-II, ASTS, PSQ-20 were performed with two time points of measurement (pretest vs. posttest). The time and time × group effects of the RM-ANOVAs are displayed in Table 2. In psychological variables, no time × group effects were found; all groups (A, B and C) improved in BDI-II, ASTS, OPD-SQS, PSQ-20 and SF-36) (see Table 3).

##### Within-Group Comparisons Pretest vs. Posttest in Psychological Variables

Depressive symptoms (measured with BDI-II) significantly decreased in the standard group (z = −2.38, *p* = 0.020) and TM group (z = 2.53, *p* = 0.010). In ASTS, positive mood significantly increased in the Yoga group (*t*(7) = 2.57, *p* = 0.037), but not in the Standard group (*t*(7) = 2.27, *p* = 0.057) and TM group (*t*(5) = 1.62, *p* = 0.165). Perceived stress (PSQ-20) decreased in the Yoga group (*t*(6) = 3.07, *p* = 0.022), but not in the Standard group *t*(5) = 2.48, *p* = 0.056) and TM group (*t*(7) = 1.90, *p* = 0.099). Sleep quality increased in the Yoga group (*t*(7) = 4.32, *p* = 0.030) but not in the Standard group (*t*(7) = −0.728, *p* = 0.490) and TM group (*t*(7) = 1.06, *p* = 0.326). Personality functioning (OPD-SQS) improved in the TM group (*t*(4) = 4.81, *p* = 0.009), but not in the Standard group (*t*(4) = 2.09, *p* = 0.105) and Yoga group (*t*(3) = 2.19, *p* = 0.116). State anxiety (STAI) and general health (SF-36) did not change within the groups (n.s.).

#### 3.4.2. Intervention Effects on Biomarkers

##### Biomarker Differences across Groups (Yoga vs. TM vs. Standard) and Time (Pretest vs. Posttest)

Cortisol significantly decreased in time after rehabilitation in all groups (see Table 4). This effect was robust and stayed significant even after Bonferroni correction. The *p*-value after Bonferroni correction for five dependent variables was set at *p* = 0.01. Nevertheless, there were no significant time × group effects for biomarkers (see Table 4). The other parameters (Trp, Kyn, Kyn/Trp ratio, neopterin) did not significantly differ between the groups (Yoga vs. TM vs. Standard) and did not significantly change over time (see Table 4). Table 5 shows the descriptive data of biomarker concentrations.

##### Within-Group Comparisons in Biomarkers

The hormonal levels displayed a non-normal distribution with many outliers. Therefore, nonparametric tests were chosen for analysis. The Wilcoxon test was used to compare the changes in participants before and after rehabilitation, and Kruskal-Wallis/Mann-Whitney tests were performed to compare changes between different groups before and after rehabilitation.

At the pretest, there was no significant difference in cortisol (Kruskal Wallis Test *H*(2) = 4.960, *p* = 0.084) between the groups. After four weeks, there was a significant difference in hair cortisol concentrations (*H*(2) = 6.314, *p* = 0.043) between the groups. Participants of standard group (standard rehabilitation) had significantly lower cortisol compared to the Yoga group (z = −2.284, *p* = 0.022), and the Yoga group had significantly higher cortisol than the TM group (z = 2.045, *p* = 0.041). After Bonferroni correction, these differences did not remain significant.

### 3.5. Correlations between Personality Functioning and Stress Markers at Baseline

Spearman correlations indicated a significant association between neopterin (at pretest) and OPD-SQS sum score (*r* = 0.53, *p* = 0.003), OPD-SQS relationship (*r* = 0.55, *p* = 0.003) and OPD-SQS self-perception (*r* = 0.53, *p* = 0.004). The correlations did not remain significant after Bonferroni correction (*p* < 0.0025)

## 4. Discussion

This interventional pilot study aimed at investigating changes in psychophysiological stress markers of patients with CVD after stress-reducing interventions in a four-week rehabilitation program. The study analyzed the effects of rehabilitation on biological stress markers such as cortisol, and on markers of the Trp/Kyn system. In addition, psychological risk factors for stress reactions, namely personality structure (OPD), were determined before and after rehabilitation.

The stress-relieving interventions of the rehabilitation program were well-accepted in the feasibility study group with high completion rates. Participants displayed substantial stress reduction after rehabilitation and, as hypothesized, improved in psychological variables (BDI-II, ASTS, OPD-SQS, PSQ-20 and SF-36). However, there were no significant group differences, suggesting an overall positive effect of rehabilitation. 

Remarkably, we observed a highly significant reduction in hair cortisol levels, and even improvement in personality functioning, after the rehabilitation intervention. As personality structure is strongly associated with stress-coping mechanisms, the improvement of personality functioning by rehabilitation is of high clinical relevance as it can improve the resilience of patients and stress relief. Cortisol significantly decreased in time in the overall samples in all groups. In addition, even personality functioning measured with OPD improved in all patients over time. Rehabilitation in the cardiovascular field is, therefore, a powerful weapon against the classic stress reaction. Cortisol has been repeatedly associated with emotional stress and cardiovascular stress. For example, a positive association has been documented between depression and acute cortisol measurements in coronary heart disease [30], as well as a trend for higher hair cortisol in older adults [31]. Chronically elevated cortisol levels are implicated in the etiology of CVD and display modulating effects on its progression and treatment [31].

Participants with impairments in personality functioning showed a significant correlation with neopterin. This is in line with our hypothesis that patients with impairments in personality functioning experience more stress. Personality functioning, also referred to as structural integration, describes basic emotion-related perception and regulation capacities directed towards the self and others. Individuals at lower levels of personality functioning typically display characteristic problems in self-regulation or self-other-differentiation (i.e., the attribution of mental states to the self or another person), accompanied by a range of challenges related to adverse health behaviors interpersonal relations, including the doctor-patient-relationship [32].

Global personality functioning has been emphasized as highly important for indication and treatment planning [33]. Our findings suggest systematic links between personality functioning and health behavior in patients with chronic diseases related to self-regulation and coping strategies. These underline the importance of assessing personality functioning for diagnosis and treatment planning in psychosomatic medicine.

Neopterin is a well-established immune activation marker with elevated concentrations seen in inflammatory states [34]. The elevation of neopterin originates from the increased synthesis by cells of monocytic origin, such as macrophages and dendritic cells, in response to interferon-gamma, which is the most central activating cytokine [35]. Neopterin suppresses migration and proliferation of vascular smooth muscle cells (VSMC) [36]. VSMC are one of the most abundant cells in vessel walls with remarkable plasticity, and have a key role in inflammation [37].

Cardiac patients with impaired personality functioning and increased stress may need information about their illness and medication; they may benefit from more frequent appointments and a more proactive attitude of the therapist or doctor. The self-awareness of patients with impairments in personality functioning should be improved by teaching them strategies and skills for regulating emotions and relationships. Long-term psychotherapy seems to be more effective in facilitating long-term changes in personality functioning [38]. Finally, they may benefit from interventions designed to foster a healthy lifestyle.

Concentrations of Trp and its metabolite Kyn did not change significantly. This result might be confounded by the small sample size, as both age and gender affect serum Trp concentrations. Nevertheless, Trp metabolites should be further investigated in CVD patients and integrated with psychoimmunological readouts. Neuroinflammation and stress-processing are substantially related, with inflammation being a key trigger of pathophysiological alterations in stress processing [39]. IDO-1 is activated by neuroinflammatory processes such as those triggered by interferon-gamma and tumor necrosis factor-alpha. Hepatic TDO regulates circulating Trp concentrations after food intake, but also responds to psychophysiological stress, e.g., if cortisol is released [40]. Thus, stress-relevant glucocorticoids and cytokines pattern affect the activity of both rate-limiting enzymes of Trp catabolism along the Kyn axis, TDO and IDO-1 [41]. The role of IDO-2 has yet to be established in this context. The increase in relevant biomarkers as metabolites of the Trp pathway for oxidative stress and chronic inflammation with activation of monocytes in the myocardium is a critical pathological process in CVD or heart failure [42]. An increased Kyn/Trp, and elevated plasma levels of 3-hydroxykynurenine and quinolinic acid are associated with increased mortality in patients with heart failure [43].

According to our hypothesis, age, sleep latency and personality functioning impact Trp, Kyn and Kyn/Trp ratio.

Participants who practiced Yoga twice a day during a four-week rehabilitation program had higher subjective and overall sleep quality improvements. The within-group comparison showed significant changes from baseline scores to the scores measured after four weeks in subjective sleep quality, sleep latency and total score. Inadequate sleep has been strongly linked to cardiovascular and metabolic diseases with alterations of biochemical and physiological functions through the regulation of the immune and endocrine system [44]. Sleep is important for maintaining inflammatory homeostasis. A lack of sleep activates the Trp pathway, leading to increased production of 3-hydroxykynurenine, quinolinic acid, and other neurotoxic metabolites [45]. Sleep deprivation triggers the activation of TDO through increase of the stress hormone corticosterone via the HPA axis, and IDO-1 through inflammatory signaling. [46]. A shift towards the Kyn route is suggested to lead to reduced Trp availability for the production of serotonin and, in consequence, of melatonin. Melatonin is involved in several processes relevant for stress regulation, such as sleep, gut and immune cell reactivity, and deficits have an impact on the whole organism [47]. Sleep, gut microbiome and the vagal nerve share a bidirectional pathway linked to stress. The vagus nerve is the primary sensory pathway by which visceral information is transmitted to the central nervous system [47]. 

Qualitative methods are increasingly used to evaluate complex interventions, such as cardiac rehabilitation, and to help understand qualitative evidence and provide information on whether and how interventions meet patients’ individual needs. Guidelines for cardiac rehabilitation in many countries recommend including educational and psychological components in addition to physical exercises [48]. Patients had diverse concerns about their cardiac disease, including fear of dying and having their lives restricted by disability or continuing treatment. This underlines the specific needs of cardiac patients and the importance of stress reduction in cardiac rehabilitation [49].

Our study demonstrates the importance of a multidimensional approach in cardiac rehabilitation along with the consequent implementation of psychophysiological measurements (such as personality functioning, depression and stress with hair cortisol measurements and Trp metabolites) to optimize the individual treatment in a personalized biopsychological therapy.

## 5. Limitations

This was a small single-center pilot study designed primarily to establish the feasibility of psychophysiological stress measurements and intervention strategies for a population of cardiac patients within their first recovery. A main limitation of this study is the small number of patients included in the study. It is remarkable that given this small number of participants, ANOVA revealed significant results regarding the cortisol level underlining the important beneficial effects of rehabilitation on stress reduction. The study may not have been adequately powered to detect statistically significant differences in many laboratory parameters. Hence, a longitudinal design could be stronger in evaluating differences in neopterin and Trp metabolism, even though all CR eligible patients were included and there were no dropouts. Our data were mainly collected from male patients. Due to the small sample size, we did not analyze the data sex specifically. However, sex is known to influence responses during perturbations [50,51]. Due to the nature of the intervention and the fact that each patient had to consent to participate in either of three groups, the allocation of patients to any group was not blinded. As a next step, we will conduct this study with a larger sample size and a longitudinal setting, including follow-up measurements. In summary, the data obtained from the current pilot study will serve as a basis for sample size calculation in further large-scale epidemiological studies.

## 6. Conclusions and Future Directions

This pilot RCT provides preliminary evidence of multicomponent rehabilitation with yoga and TM to improve cardiac patients’ short-term psychophysiological wellbeing during their first months of recovery. There was an improvement in cortisol following meditation. Future studies should consider carrying out similar studies with a larger sample size, including the same biomarkers. Stratification by impairments in personality functioning identified a subgroup of participants with worse stress perception and correlation with neopterin ratio in all groups. Future studies should examine how our results obtained in cardiac rehabilitation patients are influenced by disease states (e.g., stroke [52]) and in older persons [53,54,55,56]. Future research is needed to evaluate effects of rehabilitation on mental health, oxidative stress and CVD risk factors in a diverse patient population, with likely more impact in patients with impairments in personality functioning. These results depict a new step towards a psychosomatic research in the connection of Trp metabolism and personal stress perception, stress dysregulation and individual adaptation strategies due to personality functioning. 

## Figures and Tables

**Table 1:** Descriptive Parameters.

	Standard Group	TM Group	Yoga Group
	M (±SD)	M (±SD)	M (±SD)
Mean age (years)	58.8 (7.1)	62.1 (10.4)	59.2 (12.1)
Sex (females %)	10%	10%	50%
Follow up duration (days)	25.78 (2.71)	26.86 (0.42)	27.22 (0.41)

**Table 2:** RM-ANOVA results for psychological variables.

	Time			Time × Group		
	*F*	*p*	*η_p_* ^2^	*F*	*p*	*η_p_* ^2^
PSQI	3.39	0.099	0.274	1.53	0.268	0.253
STAI	1.21	0.300	0.119	1.75	0.229	0.280
BDI-II	7.93	0.026 *	0.531	0.471	0.643	0.119
ASTS	8.05	0.019 *	0.472	2.39	0.147	0.347
OPD-SQS	17.82	0.001 **	0.618	0.50	0.619	0.083
SF-36	5.15	0.033 *	0.183	0.15	0.866	0.012
PSQ-20	8.29	0.008 **	0.242	0.04	0.959	0.003

BDI-II = Beck-Depression Inventory-II, PSQ-20 = Perceived Stress Questionnaire, PSQI = Pittsburgh Sleep Quality Index, ATST = Aktuelle Stimmungsskala, OPD-SQS = Leven of Personality Functioning, SF-36 = Short Form Health Survey 36, STAI = 6 Item of the State-Trait Anxiety Inventory; * *p* < 0.05, ** *p* < 0.01. Significant effects after Bonferroni-adjustment in bold letters (*p* < 0.007 for *n* = 7 tests).

**Table 3:** Means and standard deviations in psychological variables from pre and post test in the groups (Standard, Yoga, TM).

		Pretest	Posttest	
		M (±SD)	M (±SD)	*p*-Value
BDI-II	Standard	7.40 (5.62)	3.40 (3.24)	0.020 *
	TM	8.60 (7.06)	4.25 (5.18)	0.010 *
	Yoga	5.00 (4.079)	4.50 (8.17)	0.128
PSQ-20	Standard	35.56 (28.02)	23.33 (20.62)	0.056
	TM	20.21 (16.58)	13.12 (11.96)	0.099
	Yoga	32.62 (16.57)	18.09 (18.03)	0.022 *
PSQI	Standard	5.57 (2.60)	6.50 (2.56)	0.490
	TM	6.50 (3.92)	4.75 (2.25)	0.326
	Yoga	7.38 (4.50)	3.37 (2.66)	0.030 *
ASTS	Standard	51.50 (17.68)	38.75 (12.09)	0.057
	TM	35.83 (12.76)	26.67 (9.24)	0.165
	Yoga	48.25 (23.01)	36.25 (16.04)	0.037 *
OPD-SQS	Standard	11.00 (3.8)	7.40 (4.27)	0.105
	TM	8.00 (4.24)	4.40 (4.56)	0.009 **
	Yoga	14.50 (6.45)	12.50 (5.26)	0.116
SF-36	Standard	56.50 (19.44)	64.50 (14.42)	0.108
	TM	65.00 (14.71)	69.28 (15.66)	0.503
	Yoga	60.00 (19.36)	66.66 (24.10)	0.141
STAI	Standard	36.29 (11.35)	29.25 (10.37)	0.199
	TM	29.62 (11.47)	25.55 (6.23)	0.374
	Yoga	35.18 (11.67)	27.40 (6.62)	0.354

Note: BDI-II = Beck-Depression Inventory-II, PSQ-20 = Perceived Stress Questionnaire, PSQI = Pittsburgh Sleep Quality Index, ATST = Aktuelle Stimmungsskala, OPD-SQS = Leven of Personality Functioning, SF-36 = Short Form Health Survey 36, STAI = 6-Item Short Form of the Spielberger State-Trait Anxiety Inventory. Differences between pre and post test: * *p* < 0.05, ** *p* < 0.01.

**Table 4:** RM-ANOVA results for biomarkers concentrations (tryptophan, kynurenine, kynurenine to tryptophan ratio [Kyn/Trp], neopterin, cortisol).

	Time			Time × Group		
	*F*	*p*	*η_p_^2^*	*F*	*p*	*η_p_^2^*
Tryptophan [µmol/L]	1.17	0.291	0.046	0.86	0.437	0.067
Kynurenine [µmol/L]	1.58	0.221	0.062	0.07	0.935	0.006
Kyn/Trp [µmol/mmol]	1.41	0.247	0.056	0.370	0.695	0.030
Neopterin [nmol/L]	0.61	0.443	0.025	0.33	0.720	0.027
Cortisol [pg/mg]	15.91	0.001 **	0.456	2.10	0.150	0.181

Note: ** *p* < 0.01, significant effects after Bonferroni-adjustment in bold letters (*p* < 0.01 for *n* = 5 tests).

**Table 5:** Descriptive data of biomarker concentrations.

		Pretest	Posttest	
		M (±SD)	M (±SD)	*p*-Value
Tryptophan [µmol/L]	Standard	55.88 (6.51)	53.88 (4.65)	0.241
	TM	53.66 (7.55)	54.37 (9.03)	0.695
	Yoga	55.54 (4.78)	54.01 (6.20)	0.297
Kynurenine [µmol/L]	Standard	2.30 (0.52)	2.31 (0.52)	0.393
	TM	2.45 (0.99)	2.32 (0.52)	0.660
	Yoga	2.32 (0.63)	2.20 (0.52)	0.446
Kyn/Trp [µmol/mmol]	Standard	41.96 (11.24)	43.33 (11.03)	0.796
	TM	46.63 (20.54)	44.03 (19.77)	0.091
	Yoga	41.91 (10.83)	40.84 (9.17)	0.743
Neopterin [nmol/L]	Standard	9.23 (3.06)	8.44 (2.06)	0.232
	TM	13.84 (10.38)	12.21 (10.28)	0.567
	Yoga	9.24 (4.05)	9.48 (3.94)	0.123
Cortisol log [pg/mg]	Standard	6.11 (0.88)	5.51 (0.79)	0.037 *
	TM	6.98 (0.64)	6.29 (0.55)	0.015 *
	Yoga	5.74 (1.07)	5.24 (0.97)	0.156

Note: Differences between pre- and posttest, * *p* < 0.05.

## Data Availability

Data is contained within article.
